# Supplementary material for: Patterns of Intron Gain and Loss in Fungi
Source: PLoS Biol. 2004 Nov 30;2(12):e422. doi: 10.1371/journal.pbio.0020422 (PMC532390; doi:10.1371/journal.pbio.0020422)
Supplement: Table S1 — Also available at http://genes.mit.edu/NielsenEtAl/. (4.3 MB ZIP). [file pbio.0020422.st001.zip › NielsenEtAl/html/161.html]

AN0813.1.NCU02617.1.MG01237.1.FG00661.1


```
 CLUSTAL W (1.82) Multiple Sequence Alignments - Introns Inserted


Sequence 1: NCU02617.1	737 aa
Sequence 2: MG01237.1	790 aa
Sequence 3: FG00661.1	751 aa
Sequence 4: AN0813.1	713 aa
Alignment Length: 793 aa
Number Identitical Residues: 318 aa
Alignment Score (without introns) 17917


MG01237.1 	MVQPANTAGAPQSLFGGGAPTAPTPGTPQGASIGAGASPSPGPGAGIGTLTASSTPSLLS
NCU02617.1	-------------MSGAGQPSIPSS-------------------------------SMTT
FG00661.1 	MAHPSTPG---QSIFGGPVAATTQTFGAGN----------------------QALTASTS
AN0813.1  	---------------------------------------------------------MAT
          	                                                           :

MG01237.1 	QTSAPGSATSSNNTFVMTNSPVKSRGIPDGYRPKVTRTLGNRPACLVNASVTYCGNNQIY
NCU02617.1	STLTPASAASSTNTYIMPNSPFKNRG--DGYRPKVTRTLGQRPACLVNASVTYCGNNQIY
FG00661.1 	LQPTPPSAASSNNTYIMPNSPAKGRSVADGYRPKVTRTLGQRPACLVNASVTYCGNNQIY
AN0813.1  	STPAPQSSLPQSAVLMPPCSKSRSRG-SAESRPSIHNVQGHVPACLVNASVTYCGNDQIY
          	   :* *: ... . : . *  :.*. .   **.: .. *: **************:***

MG01237.1 	AFGGFDQYTDEV~YNHVLRLDLVSLQWSLVDNYGDIPGVRMG1HTATLYKGDRLLVFGGE
NCU02617.1	AFGGFDQYTDEV~YNHVLRLDLVSHQWSLVDNYGDIPGVRMG1HTATLYKGDKLLVFGGE
FG00661.1 	AFGGFDQYTDEV~YNHVLRLDLVSHQWNLVDNYGDIPGVRMG1HTATLYQGNKLLVFGGE
AN0813.1  	AFGGFDQYTDEV1YNHVLRLNLKTLRWDLVDNYGDIPGVRMG1HTANLYQDNKLIVFGGE
          	************ *******:* : :*.************** ***.**:.::*:*****

MG01237.1 	NEHRTYLSDLIIFDLKTAHWTQPQCSGPIPRGRARHAAILHEDKLFIVGGITGHDNHVLD
NCU02617.1	NEHRTYLSDLIIFDLKTAHWTQPQVTGPIPKGRARHAAVLHQDKLFIIGGITGHDNYVLD
FG00661.1 	NEHRTYLSDLIVFDLKTAHWTQPLVSGPIPKGRARHAAVLHEDKLFVIGGITGQNNYVLD
AN0813.1  	NEHGEYLSDVIIFDVPTSTWTQPEVRGQVPRGRARHAAVIHEDKLFILGGSESSG--ILD
          	***  ****:*:**: *: ****   * :*:*******::*:****::**  . .  :**

MG01237.1 	DVCFLDLKTFTWSRSWRFVSRFDHSASIW2NDRVWVYGGLSEEMNRTSELWWLDLKGSPA
NCU02617.1	DICYLDLKTFTWSRSWRFVGRFDHSAYIW~GERVWVFGGLSEEMDKVSDIWWLDLKGSPA
FG00661.1 	DICYLDLKTFTWSKSWRFVGRFDHSAYIW~GDRVWVFGGLSEDMDKISDLWWLDLKGSPQ
AN0813.1  	DMFYLDLKTWTWSRPWKFTARFDHLAWVW~GGRLWTFGGLDPDMEKTTDIWWLDLQAIPA
          	*: :*****:***:.*:*..**** * :* . *:*.:***. :*:: :::*****:. * 

MG01237.1 	FETPPQVGTYDRASLASRVSHSARPASYSPLVQSPIVGTSGYAANSRTAQVNPPSFQLKS
NCU02617.1	FDSPPQIGSMDRSGMA-RSAISPRPS--YPAAQSPIVGSSGYAANSRTAQVNPPSFHLKT
FG00661.1 	FDSRPQMGLFDRGSMSTRASNSPRPP--YTMPQAPVVGASGYAANSRTAQVNPPSHQLKT
AN0813.1  	SGTPDHQGTMDTPARIGSAAHSLDNNIYSNMGQQLPGRSGSYAANSASVQVRNASRRKS-
          	  :  : *  *  .     : *          *     :..***** :.**. .* : . 

MG01237.1 	FAPIAPGTISSIKFHSGPIVPEQGTGYHFHVYSSGTLLDFLTPTTSSSSKDCSLSALDLN
NCU02617.1	YAPPAPGAVSALKFVNGPNVPSQGQGIHFHVYSSGTLLDFVTPAATISSKECSLSALDLG
FG00661.1 	YAPLAPGSISALKFMSGPSIPSQGSGLHYHAYSSGTLLDFVTPAATLTSRECSLSALDLG
AN0813.1  	---IAPGSISCLQFKSGPNVPSLFSGTHFQAFASGVLLDLITPSETTPIRDCNLSSLELD
          	    ***::*.::* .** :*.   * *::.::**.***::**: : . ::*.**:*:*.

MG01237.1 	SLRWQKLAEGREIFKSGYRWHYCTLSEDGTKAWLLGCPSEQSALDLGANGLEEYLS~DIM
NCU02617.1	SLRWQKLADGREIFKPGYRWHYCTMNEDGTKAWLLGCPTEPNVLDHGGTGLEEYLS~DIM
FG00661.1 	TLRWTKLAGGREIFKAGYRWHYCCMNEDGTKSWLLGCPTDPAATDIGPNGYEEYLS2DIM
AN0813.1  	SARWQKLVDGQEIFRPGYRWHYLTVDTSGTKAWLLGCSLDP--GNTPGSGDENHMS~QVL
          	: ** **. *:***:.******  :. .***:*****. :    :   .* *:::* :::

MG01237.1 	EVDLRRYGFLGNNMAPEPRAELLRPSSRIRIVEQPSRGLGADLATLFNVPPEAGSGTDFV
NCU02617.1	EIDLRRYGFLGNNFAPEPRADFTRPSSRARHIEQPSKGLGCDLAKLFDQPPETGSGTDFI
FG00661.1 	EIDLRRYGFLGDNLTPEPRSE-SRPSATTRAMDQPSKGLGSDLAKLFNQPPETGSGTDFI
AN0813.1  	SIDLERYGLLGNEMS-ALSPDPRKAAVLGRAQVGPLSGLGADLSTAFDRPPESGSGADFT
          	.:**.***:**::::    .:  :.:   *    *  ***.**:. *: ***:***:** 

MG01237.1 	VTALADDCEDESMVGSALVQPSDSSSVGGADSWLSPDAPTSSPIYVHRLILQARWPHFRR
NCU02617.1	VTALSREYDEDEIIGSALVHTSDS--MDGDQTWLSPDAPTSPPIHVHRLILQARWPHFAR
FG00661.1 	ITALAGDYEDDDTLTSSMTRNDET---SQNQSWLSADAPTSQPIHVHKLILQARWPHFAR
AN0813.1  	ITANS----DDHIYTDSNAEDGFS--ERSTSTFLPENAATSPPIHVHKLILQLRWPHFKR
          	:** :    ::    .: .. . :      .::*. :*.** **:**:**** ***** *

MG01237.1 	LWASQMAEFHTRKMHIPEPYSVVKAFLYYLYTDRIDPSIEDEGADSGTTTDLSDIAGLLV
NCU02617.1	LWSSQMAEFHTKKMHIPEPYSVVKAFLYYLYTDRIDPGSEE---DESITADLSDVAGLLV
FG00661.1 	LYNAQMAEFHTKKMHIPEPYSVVKAFLYYLYTDSIRGTSDS---DSDATTALSDVAGLLV
AN0813.1  	LYSAQMVEYHTNRMHIPEPYSVVRAFLYYLYTDSIAGHPEY-------CADIIDVAGMLV
          	*: :**.*:**.:**********:********* *    :         : : *:**:**

MG01237.1 	MSNIYGIPHLRLLCVNRLAKELDVDHACIIWYRAGLADEEWLRKRAAAYCLTHWGRIVRT
NCU02617.1	MSNIYNIPHLRLLCVNRLAKELDVDHACIIWYCAGMANEEWLRKRAATFCMTHWGRVVRT
FG00661.1 	MSNIYNIPHLRLLCVNRLAKELDVDHACIIWYCAGLANEEWLRKRAAGFCMIHWGRVVRT
AN0813.1  	MANLYDLPRLRLLCVSRLGRELDVENAAIIWDRAGRTNEEWLMRRAAQFCLNHWGRVVRT
          	*:*:*.:*:******.**.:****::*.***  ** ::**** :*** :*: ****:***

MG01237.1 	SGFLRLPRSALVELSQEIDMEGRVIGGEELDMFVGST-MATDSSGALARRKESMSSQPSQ
NCU02617.1	LGFQRLPRAALVELSQEVDMEGRVVGGDELDLMVGIDGRYHDGGSGSSRRKGSISSNHTQ
FG00661.1 	QGFVRLPRTALVELSQEVDMEGRVIGGEELEWGGMNDSMGHDA------RKESISSNQTQ
AN0813.1  	DGFRSLSRESIIDLCEVVDMEGRIVAGPELELVGTLGADDGLGSGRELKRSSQLTLNGVT
          	 **  *.* ::::*.: :*****::.* **:      .    ...    *. .:: :   

MG01237.1 	MIESEVDDDDGMELN--
NCU02617.1	VLESELDEDEGMEMS--
FG00661.1 	TIEDDGDDDEGMEMN--
AN0813.1  	LDGDEADADEMDGVEDI
          	   .: * *:   :..
```
